# Safety and anti-HIV assessments of natural vaginal cleansing products in an established topical microbicides *in vitro *testing algorithm

**DOI:** 10.1186/1742-6405-7-22

**Published:** 2010-07-09

**Authors:** Carol S Lackman-Smith, Beth A Snyder, Katherine M Marotte, Mark C Osterling, Marie K Mankowski, Maureen Jones, Lourdes Nieves-Duran, Nicola Richardson-Harman, James E Cummins, Brigitte E Sanders-Beer

**Affiliations:** 1 Southern Research Institute, Frederick, MD, USA; 2 Alpha StatConsult LLC, Damascus, MD, USA; 3 Division of AIDS/NIAID, National Institutes of Health, Bethesda, MD, USA; 4 BIOQUAL, Inc., Rockville, MD, USA

## Abstract

**Background:**

At present, there is no effective vaccine or other approved product for the prevention of sexually transmitted human immunodeficiency virus type 1 (HIV-1) infection. It has been reported that women in resource-poor communities use vaginally applied citrus juices as topical microbicides. These easily accessible food products have historically been applied to prevent pregnancy and sexually transmitted diseases. The aim of this study was to evaluate the efficacy and cytotoxicity of these substances using an established topical microbicide testing algorithm. Freshly squeezed lemon and lime juice and household vinegar were tested in their original state or in pH neutralized form for efficacy and cytotoxicity in the CCR5-tropic cell-free entry and cell-associated transmission assays, CXCR4-tropic entry and fusion assays, and in a human PBMC-based anti-HIV-1 assay. These products were also tested for their effect on viability of cervico-vaginal cell lines, human cervical explant tissues, and beneficial *Lactobacillus *species.

**Results:**

Natural lime and lemon juice and household vinegar demonstrated anti-HIV-1 activity and cytotoxicity in transformed cell lines. Neutralization of the products reduced both anti-HIV-1 activity and cytotoxicity, resulting in a low therapeutic window for both acidic and neutralized formulations. For the natural juices and vinegar, the IC_50 _was ≤ 3.5 (0.8-3.5)% and the TC_50 _≤ 6.3 (1.0-6.3)%. All three liquid products inhibited viability of beneficial *Lactobacillus *species associated with vaginal health. Comparison of three different toxicity endpoints in the cervical HeLa cell line revealed that all three products affected membrane integrity, cytosolic enzyme release, and dehydrogenase enzyme activity in living cells. The juices and vinegar also exerted strong cytotoxicity in cervico-vaginal cell lines, mainly due to their acidic pH. In human cervical explant tissues, treatment with 5% lemon or lime juice or 6% vinegar induced toxicity similar to application of 100 μg/ml nonoxynol-9, and exposure to 10% lime juice caused tissue damage comparable to treatment with 5% Triton-X-100.

**Conclusions:**

Lemon and lime juice and household vinegar do not fulfill the safety criteria mandated for a topical microbicide. As a result of their unphysiological formulation for the vaginal tract, they exhibit cytotoxicity to human cell lines, human vaginal tissues, and beneficial vaginal *Lactobacillus *species.

## Background

Human immunodeficiency virus (HIV) infection and the resulting clinical disease, AIDS, has continued to be a world-wide epidemic since its discovery in 1982 [1,2]. Despite extensive international research efforts and funding support, no effective preventive measures for HIV apart from behavioral modifications and condom use have been shown fully effective to date [3]. Some of the research community has shifted its attention to the development of topical microbicides, defined as substances that prevent the sexual transmission of infectious agents [4]. Five chemical products were advanced to Phase III clinical trials, but all were discontinued due to either toxicity or lack of efficacy [5,6]. Although the medical, scientific, and regulatory compliance communities in industrialized countries foster the use of commercially purchased and chemically defined drug substances for HIV prevention and a success-by-design drug development strategy, the situation in low resource settings is remarkably different. In countries with poorly regulated and minimally subsidized health care systems, access to effective HIV prevention methods is a direct consequence of individual financial wealth and/or community-wide, cultural acceptability. In the context of prevention, women with no monetary assets may not have access to the newest technologies and thus develop their own strategies, often inspired by community shamanism or non-peer reviewed information from public media, such as newspaper articles.

One of the oldest, least expensive practices for genital cleansing has been the application of commonly available food products, since they are easily accessible and require little or no pre-use preparation [7]. For example, lemon and lime juices have historically been introduced into the vagina to prevent pregnancy or sexually transmitted diseases [8-11], The contraceptive properties of lemon and lime juice were scientifically validated since it was shown that the acidic pH in lemon-based drinks decreases sperm motility [12].

Lemons and limes have a similar chemical content and are primarily composed of water and 5% citric acid, giving these fruits a tart taste. Other components are maleic acid, ascorbic acid (vitamin C), various ions, enzymes, and flavonoids [13-15]. Carbohydrates, in the form of simple sugars and polysaccharides, comprise most of the soluble solids in citrus fruits. The citrus flavor is due to a blend of sugars, acids, and specific flavor compounds, some of which are sugar-containing substances known as glycosides. Contribution to fruit color is made by sugar-containing anthocyanidins, while texture is controlled by the structural carbohydrate polymers. The low pH just above 2 results from the high acid content. In contrast to lemons and limes, white distilled household vinegar is more defined in its chemical composition. It is made from selected sun-ripened grain and diluted with water to a uniform pickling and table strength of 5% (50 grains) acidity. Undiluted vinegar also has a pH just above 2, similar to that of lemon and lime juice.

In June 2004, 56% of 300 sexually active Nigerian women interviewed reported use of vaginal lemon/lime juice douches used neat or diluted in water before or after sex [8]. Based on this knowledge both preclinical and clinical safety evaluations were undertaken to determine the clinical benefit of this practice. Lime juice was found to be virucidal to HIV-1 and cytotoxic to cervico-vaginal epithelial cells [16]. In another report, the high acidity of lemon juice appeared to be responsible for the loss of viability of vaginal cells and *Lactobacillus *species [17,18].

The present study focuses on a comprehensive assessment of the use of natural and neutralized lime and lemon juices and white household vinegar in a highly standardized *in vitro *testing algorithm that is currently supported by the National Institute of Allergy and Infectious Diseases (NIAID) to identify potential microbicide candidates. This algorithm includes established cell-based HIV-1 transmission assays, human cervical tissue explant assays, and *Lactobacillus *toxicity tests [19].

## Results

### Natural Lime and Lemon Juice and Household Vinegar are Toxic to Immortalized and Primary Cells, and Toxicity is Reduced by pH Neutralization

**Figure 1:**
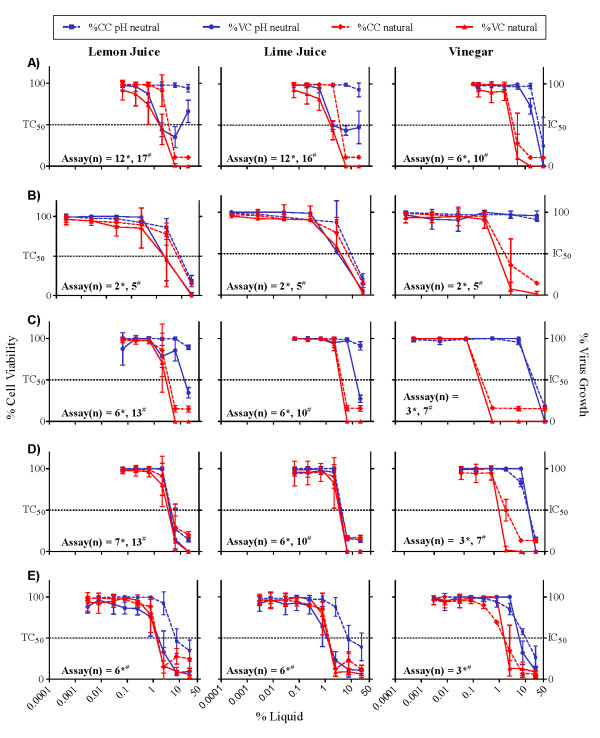
**HIV-1 Replication Inhibition and Cytotoxicity with Increasing Concentrations of Lemon Juice, Lime Juice, and Vinegar in Cell-based Assays**. CCR5-tropic cell-free HIV-1 entry assay (A), CCR5-tropic cell-associated HIV-1 transmission assay (B), CXCR4-tropic cell-free HIV-1 entry assay (C), CXCR4-tropic fusion assay (D), and PBMC antiviral assay (E). Virus growth (shown as % of virus control (VC), solid lines) and cell viability (shown as % viability of untreated cell control (CC), dashed lines) are presented for lemon juice, lime juice, and vinegar. Results are shown for both pH neutral (blue) and natural (red) formulations. Means ± standard deviations (SD) of replicate experiments are presented. The black, horizontal line indicates the level for 50% cell death (i.e., TC_50_) or 50% virus inhibition (i.e., IC_50_), respectively, for each assay. The number of experiments performed (n) is indicated within each figure for the pH neutralized (*) and the natural products (#). The concentration of juice or vinegar is expressed as percent (%) solution (v/v).

The antiviral and cytotoxic effects of natural and pH neutralized lemon and lime juices and house-hold vinegar in cell-free and cell-associated HIV-1 transmission inhibition assays are presented in Figure 1a-e. In the CCR5-tropic and CXCR4-tropic cell-free HIV-1 entry assays (Figure 1a and Table 1), the IC_50_s and TC_50_s of natural lemon and lime juices and vinegar ranged from 1.7 to 4.1% solution (v/v), resulting in a very low therapeutic index (0.9-2.3). Neutralization of the juices increased the therapeutic index for lemon and lime juice in the CCR5-tropic assay, but not in the CXCR4-tropic assay. Neutralization of vinegar abolished both efficacy and toxicity in the CCR5-and CXCR4-tropic HIV-1 entry assays. For the CCR5-tropic cell-associated HIV-1 transmission assay, the CXCR4-tropic fusion assay, and the HIV-1 PBMC assays the therapeutic indices remained low (≤ 7.0) whether the juices and vinegar were neutralized or not. The therapeutic index was especially low in the HIV-1 fusion assays, where HeLa cells were exposed to the juices and vinegar for 48 hours.

**Table 1:** IC_50_, TC_50_, and Therapeutic Index (TI) for Lemon, Lime, and Vinegar in Various Cell-based HIV-1 Assays

		Lemon Juice			Lime Juice			Vinegar		
		
		IC_50_	TC_50_	TI	IC_50_	TC_50_	TI	IC_50_	TC_50_	TI
	**Unit**	**%**	**%**	**-**	**%**	**%**	**-**	**%**	**%**	**-**

**CCR5-tropic Cell-free HIV-1 Entry Assay**	Natural	1.8^1 ^(0.9)	3.8 (0.2)	2.1 (1.7)	1.7 (1.1)	3.8 (0.1)	2.3 (1.9)	2.9 (0.6)	3.0 (0.0)	1.1 (0.2)
	
	pH Neutral	1.7 (0.3)	> 20.0 (0.0)	**>**11.8 (2.2)	2.0 (0.6)	> 20.0 (0.0)	> 10.2 (2.8)	23.3 (4.2)	29.2 (2.0)	1.2 (0.3)

**CCR5-tropic Cell-associated HIV-1 Transmission Assay**	Natural	2.0 (3.7)	6.3 (0.3)	3.2 (5.7)	2.4 (2.0)	6.2 (0.6)	1.5 (1.5)	0.8 (0.1)	1.0 (0.8)	1.3 (0.9)
	
	pH Neutral	2.7 (3.0)	8.5 (0.6)	4.7 (5.6)	4.9 (7.4)	9.0 (1.0)	4.2 (6.1)	> 25.0 (0.0)	> 25.0 (0.0)	1.0 (0.0)

**CXCR4-tropic Cell-free HIV-1 Entry Assay**	Natural	3.5 (0.4)	4.0 (0.5)	1.2 (0.2)	3.5 (0.5)	4.1 (0.3)	1.2 (0.1)	3.2 (0.2)	3.2 (0.0)	0.9 (0.1)
	
	pH Neutral	14.1 (2.9)	> 20.0 (0.0)	> 1.4 (0.3)	13.9 (0.7)	> 20.0 (0.0)	> 1.4 (0.1)	32.4 (2.0)	31.0 (1.6)	1.0 (0.1)

**CXCR4-tropic Fusion Assay**	Natural	3.4 (0.7)	4.3 (0.3)	1.2 (0.1)	3.4 (0.7)	4.1 (0.8)	1.2 (0.2)	1.7 (0.1)	2.4 (1.5)	1.5 (1.0)
	
	pH Neutral	3.7 (0.2)	4.6 (0.3)	1.2 (0.1)	3.5 (0.3)	4.3 (0.2)	1.2 (0.1)	17.4 (1.2)	13.8 (0.7)	0.8 (0.1)

**PBMC Antiviral Assay**	Natural	1.3 (0.5)	1.4 (0.3)	1.1 (0.2)	1.4 (0.8)	1.4 (0.4)	1.1 (0.5)	1.2 (0.4)	1.0 (0.2)	0.7 (0.21)
	
	pH Neutral	1.8 (1.7)	6.7 (8.1)	4.0 (9.0)	1.2 (0.7)	10.6 (17.8)	7.0 (13.5)	4.8 (2.0)	9.6 (3.2)	1.7 (0.4)

^1^Median and inter-quartile range

Juice and vinegar concentrations are expressed as percent (%) solution.

Lemon and lime juice and vinegar were also tested in the presence of 25% pooled human seminal plasma in the CCR5-tropic cell-associated HIV-1 transmission assay, but the addition of the alkaline seminal plasma did not result in any changes to the efficacy, toxicity, or the therapeutic index of the three liquids (data not shown).

### Freshly Processed Lemon and Lime Juice and Household Vinegar are Toxic to Beneficial *Lactobacillus *Species Commonly Found in the Human Vaginal Tract

**Figure 2:**
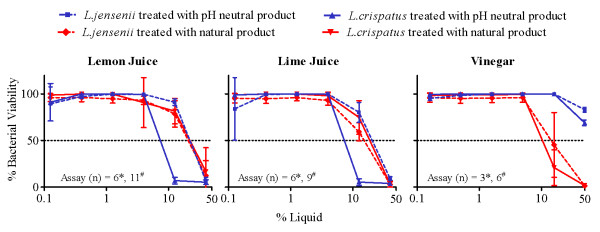
**The Effect of Increasing Concentrations of Lemon Juice, Lime Juice, and Vinegar on Viability of *L. crispatus *and *L. jensenii***. Percentages of bacterial viability after treatments with different concentrations of neutralized (blue) or natural (red) lemon juice, lime juice, or vinegar (compared to untreated control) are presented. Results are shown for *L. crispatus *(solid lines) and *L. jensenii *(dotted lines) assays. Means ± standard deviations (SD) of replicate experiments are presented. The number of experiments performed (n) is indicated within each figure for the pH neutralized products (*) and natural products (#). The concentration of juice or vinegar is expressed as percent (%) solution (v/v).

Both juices and vinegar demonstrated strong antimicrobial activity against *Lactobacillus jensenii *and *L. crispatus *(ATCC 25258 and 33820, respectively, Table 2 and Figure 2); the MIC_50_s ranged from 12.1% to 18.4% for *L. jensenii *and from 9.9% to 19.6% for *L. crispatus*. Neutralization of vinegar, but not of lemon and lime juice removed toxicity to beneficial *Lactobacillus *species. *L. crispatus *appeared to be more susceptible to the higher pH condition as demonstrated by lower viability in the presence of 12.5% neutralized lemon or lime juice (p < .0001; Figure 2). No significant differences between the natural and pH neutral juices were noted at any other concentrations tested in *L. crispatus *(Figure 2).

**Table 2:** Effect of Lemon and Lime Juice and Vinegar in *Lactobacillus *Toxicity Assays

		**Lemon Juice**	**Lime Juice**	**Vinegar**
		
***L jensenii***	Natural	18.4 (1.2)^1^	14.0 (3.2)	12.1 (13.0)
	pH Neutral	22.0 (0.7)	21.1 (2.9)	> 50.0 (0.0)
***L crispatus***	Natural	19.6 (3.6)	18.0 (6.6)	9.9 (2.4)
	pH Neutral	7.6 (0.3)	7.8 (0.6)	> 50.0 (0.0)

^1^Median and inter-quartile range of MIC_50_s

Juice and vinegar concentrations are expressed as percent (%) solution.

### Comparison of Three Different Toxicity Endpoints in the Cervical HeLa Cell Line Revealed that Lemon and Lime Juice and Vinegar Affect Membrane Integrity, Cytosolic Enzyme Release, and Dehydrogenase Enzyme Activity in Living Cells

Toxicity of lemon and lime juice and household vinegar was tested in three assays that measure different cytotoxicity endpoints. Lemon and lime juice and household vinegar exerted ≥ 50% loss of cell viability at concentrations of 4%, demonstrating damage to the cell membrane and cytosol. The TC_50_s in all three assays ranged from 3.8 to 4.8% solution for the three liquid food products with very little variability (Table 3). Neutralization of these products removed the cytotoxic effects.

**Table 3:** Cytotoxicity of Lemon and Lime Juice and Vinegar in MAGI-CCR5 Cells following a 3 Hour Exposure using Different Toxicity Endpoints

		Toxicity Assay Method		
		
		Mitochondrial^1^ Reduction of MTS	Membrane Integrity^2^	Cytosolic Enzyme Release^3^
**Test Article**	**Unit**	**TC_50_**		

Triton X-100	%	0.02	0.02	0.02
AMD 3100	μM	> 10	> 10	> 10
Lemon Juice, natural	%	4.0 ± 0.4^4^	4.5 ± 1.0	4.4 ± 0.3
Lemon Juice, neutral pH	%	> 20	> 20	> 20
Lime Juice. Natural	%	3.8	4.1	4.6
Lime Juice, neutral pH	%	> 20	> 20	> 20
Vinegar, natural	%	3.8	4.2	4.8
Vinegar, neutral pH	%	> 20	> 20	> 20

^1^CellTiter 96^® ^AQueous One Solution Cell Proliferation Assay (MTS) (Promega)

^2^Live/Dead^® ^Assay for Cell Viability (Invitrogen)

^3^Vybrant™ Cytotoxicity Assay (Invitrogen)

^4^Data for these assays are reported for a single experiment with 3 replicates, except for the assays with natural lemon juice where the experiment was performed three times and thus the standard deviation is shown.

Juice and vinegar concentrations are expressed as percent (%) solution.

### Freshly Squeezed Lemon and Lime Juice and Household Vinegar Exhibit Strong pH-Dependent Cytotoxicity to Cervico-Vaginal Cell Lines

**Figure 3:**
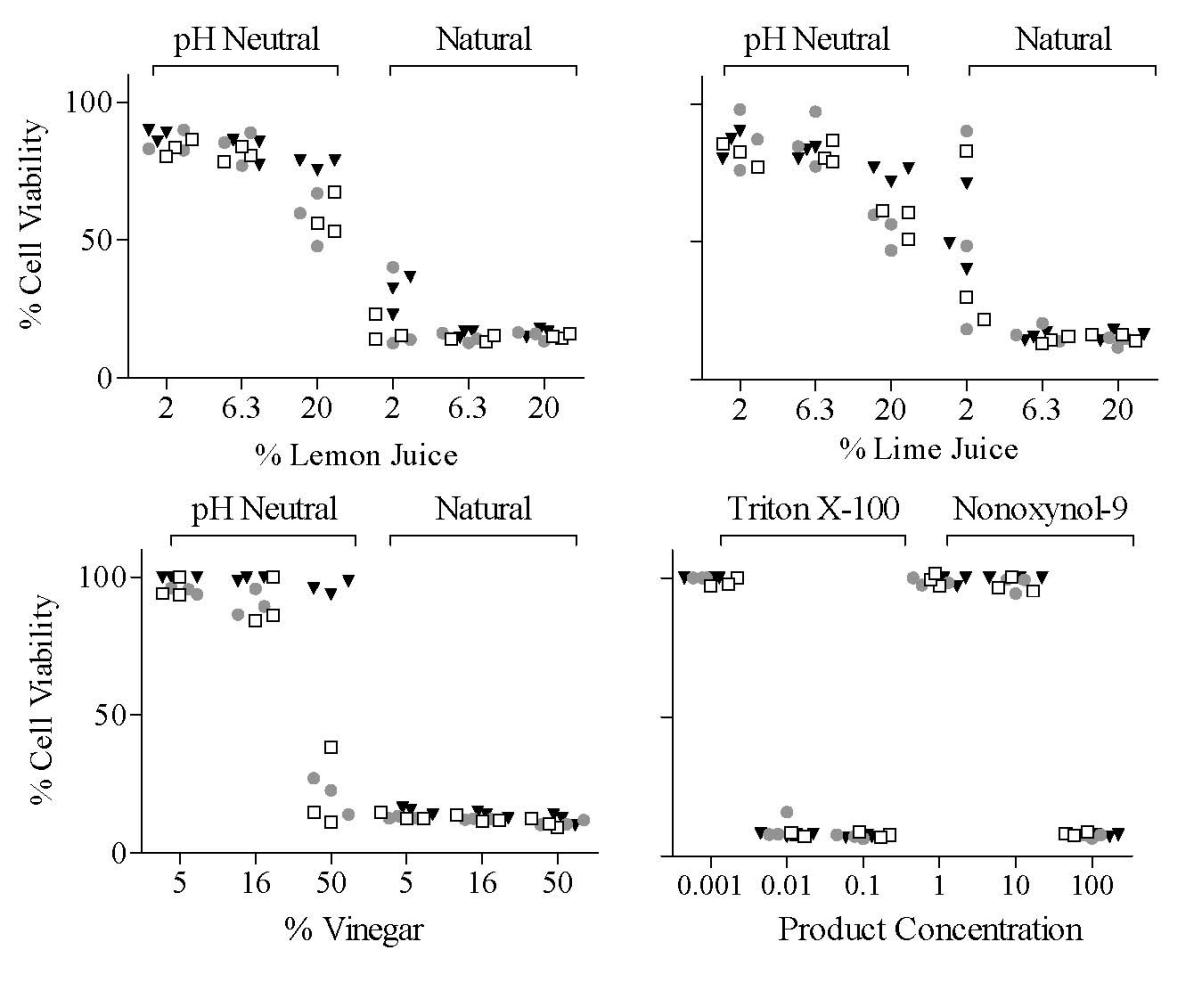
**Effect of Lemon Juice, Lime Juice, Vinegar, Nonoxynol-9, or Triton X-100 Treatments on Viability of Cervico-vaginal Cell Lines**. Percent cell viability (compared to untreated control) after treatment with (a) 2, 6.3, or 20% lemon or lime (pH neutral or natural) juices; (b) 5, 16, or 50% vinegar (pH neutral or natural); (c) 0.001, 0.01, or 0.1% Triton X-100, and (d) 1, 10, or 100 μg/mL nonoxynol-9 was measured for ECT1 (clear squares, n = 3), END1 (grey circles, n = 3), and VK2 (black triangles, n = 3) cell lines. Each data point represents the average of 3 replicates as described in the methods. The concentration of juice or vinegar is expressed as percent (%) solution (v/v).

In order to determine if the toxic effects observed for permanent cell lines and PBMC would be similar in cells derived from cervical and vaginal tissues, cell viability was assessed following exposure to natural and neutralized lemon or lime juice and vinegar (Figure 3). The effect of the juices on viability of ectocervical, endocervical, and vaginal cell lines was consistent across cell types, with 6.3-20% solutions of freshly prepared juices exerting toxic effects on all cell types. Neutralized juices at these concentrations caused much less toxicity in these cell lines. The ectocervical and endocervical cell lines appeared to be more affected by the cytotoxic effects of 20% pH neutral juices compared to the effect in vaginal cells, and this effect was much more dramatic for vinegar at the 50% concentration. Triton X-100 and nonoxynol-9 data are shown for comparative reasons to illustrate their effects on cell viability, since these substances have been reported to exert cytotoxic activity [20].

### Lemon and Lime Juices Demonstrate Similar or Greater Toxicity than Triton X-100 and N-9 in Human Cervical Explant Tissue

**Figure 4:**
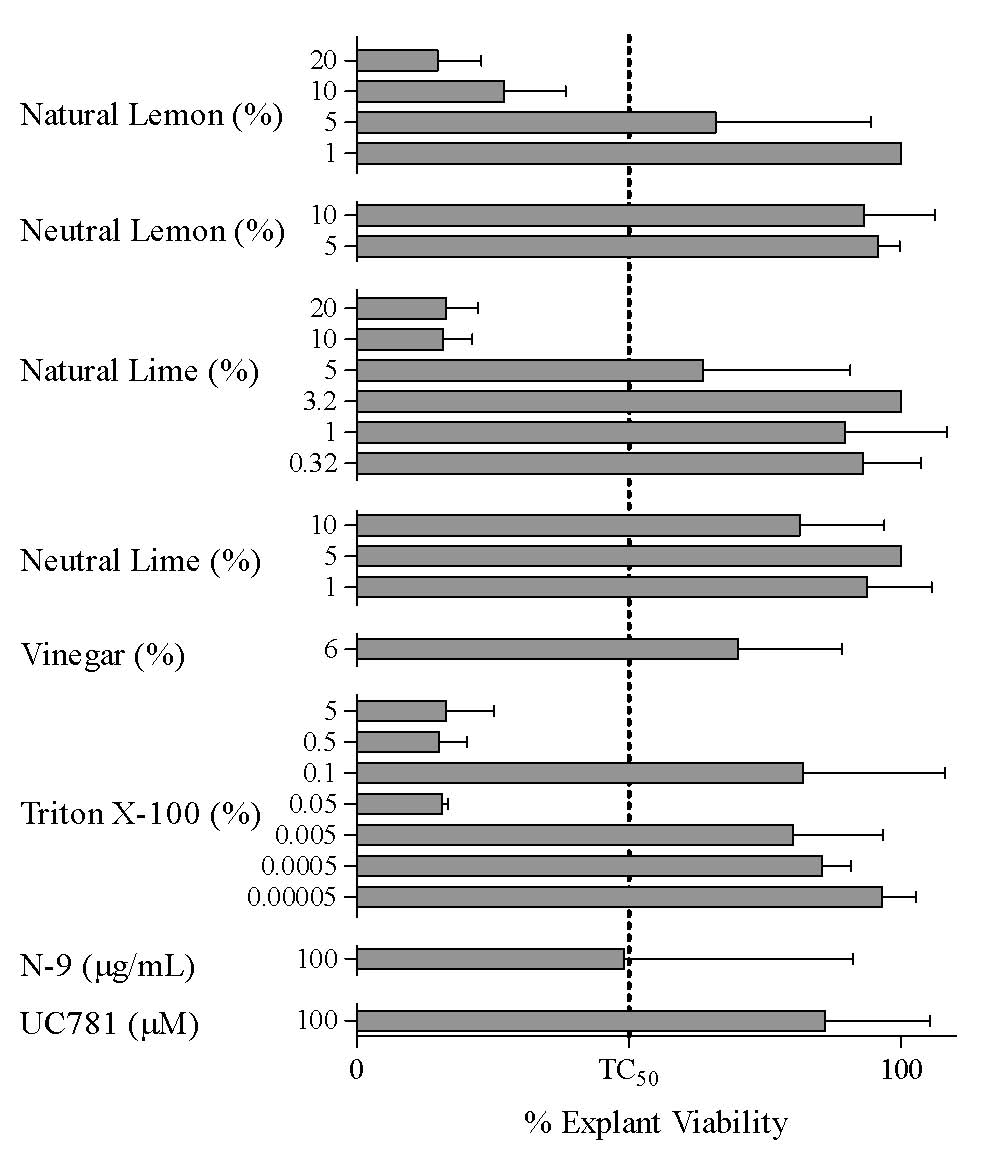
**Effect of Lemon Juice, Lime Juice, Vinegar, Nonoxynol-9 (N-9), and Triton X-100 on Viability of Cervical Explant Tissues**. Effects of lemon juice, lime juice, vinegar, Triton X-100, nonoxynol-9 (N-9), and UC781 on viability of cervical explant tissues. Presented are the percent viability for tissues treated with lemon juice (1-20%), lime juice (0.32-20%), vinegar (6%), Triton X-100 (0.00005-5%), nonoxynol-9 (N-9; 100 μg/mL), and UC781 (100 μM), compared to donor-matched, untreated controls (defined as 100%). Tissues were exposed from 2 hours to overnight. Each bar represents data from 1 to 6 donors. Bars indicate mean ± SD for each product/concentration. The concentration of juice or vinegar is expressed as percent (%) solution (v/v).

Since lemon and lime juice and vinegar were toxic to primary and transformed cell lines of various origin and vaginal *Lactobacillus *species, the next goal was to assess the cytotoxicity of these liquids in freshly obtained human cervical tissues (Figure 4). Human cervical explant tissues were exposed to the juices, vinegar, N-9, and Triton X-100, and the percent viability of the tissues is shown in Figure 4. The % viability of exposed tissues was higher for N-9 at 100 μg/mL than for the juices at ≥ 10%. Ten percent lemon juice reduced tissue mean viability by > 70%, and 10% lime juice reduced viability by > 80% as compared to tissue treated with culture medium only (Figure 4). Exposure of tissue to N-9 (100 μg/mL) or 0.3% acetic acid (6% household vinegar) reduced tissue viability by 50 and 30%, respectively. Five percent lemon or lime juice appeared less toxic than the 10% concentrations showing a clear dose-response effect on the tissues. Table 4 summarizes the anti-HIV-1 effect of lemon and lime juice compared to the untreated HIV-1 infected control in explant tissue. Virus replication was determined as a function of HIV-1 p24 in culture supernatants [21]. There was little or undetectable HIV-1 replication in the 5-20% lemon and lime juice treated samples compared to the untreated HIV-1 infected control, where an average of 3,090 pg/mL HIV-1 p24 Gag was measured. Treatment with 1% natural juice or neutralized juices up to 10% resulted in virus replication levels comparable to that of untreated virus controls.

**Table 4:** Summary of Antiviral Effects of Lemon and Lime Juice in Cervical Explant Tissues

Test Article	Unit	Concentration	p24 Log_10_^1^	# of donors
Lemon Juice, natural	%	1	2.75	1
	%	5	0^2 ^± 0	3
	%	10	0.36 ± 0.5	2
	%	20	0	1

Lemon Juice, neutral pH	%	5	3.23	1
	%	10	2.46 ± 0.13	2

Lime Juice, natural	%	1	2.61 ± 0.16	2
	%	5	0 ± 0	3
	%	10	0.32 ± 0.55	3

Lime Juice, neutral pH	%	1	3.26 ± 0.32	2
	%	5	3.53	1
	%	10	2.22 ± 1.94	3

Untreated HIV-1 Infected Control			3.49 ± 0.54	11

^1^Data are expressed as the mean ± standard deviation log_10 _of HIV-1 p24 in each sample.

^2^0 = Undetectable p24 protein

Juice and vinegar concentrations are expressed as percent (%) solution.

### Antiviral and Cytotoxic Effects of Other Juices Tested in Cell-based Assays

To determine if other fruit or vegetable juices possessed antiviral or cytotoxic properties, tomato, grapefruit, orange, and apple juices were evaluated at their natural pH and in neutralized form for antiviral and cytotoxic effects in the CCR5-tropic cell-associated HIV-1 transmission assay. The results for freshly prepared grapefruit juice (pH 2.95) were similar to the antiviral and cytotoxic effects seen for lemon and lime juice, and pH neutralization eliminated any antiviral and cytotoxic effects up to the highest concentration tested (25%). Apple, orange, and tomato juice (natural pH 3.7, 3.3, and 3.9, respectively) did not demonstrate any cytotoxic or antiviral effects at the highest concentration tested (25%) whether they were natural or neutralized (data not shown).

## Discussion

In this study, lemon and lime juice and vinegar were tested in a variety of cell-based assays that are routinely used to evaluate compounds for their potential development as topical microbicides to prevent the sexual transmission of HIV. It is important to note that this is the first study, in which multiple natural products, all commonly used for vaginal cleansing, were evaluated in parallel in a highly standardized *in vitro *algorithm. In each assay where natural lemon or lime juice was used, the cytotoxicity observed dominated the activity profiles of the juices, providing minimal separation of antiviral efficacy from nonspecific cytotoxic effects on cell lines, primary cells, and explant tissue.

HIV-1 entry assays demonstrated inhibition of virus replication whether the pH of the juices was acidic or neutral, suggesting that the juices possess antiviral effects from some uncharacterized component that is pH-independent. Further, the acidity of the juices did not appear to be responsible for antiviral activity in the cell-associated transmission assay, based on the observation that natural and neutralized juices were equally inhibitory. This is in contrast to results obtained using vinegar, where removal of the acidity also removed the antiviral and cytotoxic effects. Although the cytotoxic effects of the juices in the entry and transmission assays seemed to decrease or disappear following neutralization, it should be noted that the nature of these assays (removal of virus and inhibitor after 3-4 hr incubation followed by 24-48 hr incubation in the absence of inhibitor) allows the cells to recover. The effect of continuous exposure of the juices to the cells for 2 days is seen in the CXCR4-tropic fusion assay where no wash-out occurs. Here, the cytotoxic effects of the juices are indistinguishable from the antiviral fusion effects, regardless of pH. This demonstrates that the toxicity of natural juices is severe following a short or long exposure, as also reported by Fletcher et al. [16] In contrast in the fusion assay, neutralization of vinegar reduced both antiviral and cytotoxic effects. Taken together, this suggests that the toxicity observed from exposure to the juices is also not solely pH-dependent.

Consistent with observations in cell-based assays, in cervical explant tissues most of the anti-HIV-1 activity of lemon and lime juice appeared to result from necrosis of HIV-1 target cells, again implying a low therapeutic window for application of these citrus juices *in vivo*. Here, 10% lemon or lime juice exerted more toxicity in cervical tissue than did nonoxynol-9 (N-9, 100 μg/mL), a spermicide that was the first microbicide candidate evaluated in clinical trials (at a dosage of 52.5 mg). It was subsequently withdrawn from clinical testing as a result of increased risk of sexually transmitted infection due to disruptions in the vaginal and rectal epithelium following repeated exposure [22]. In addition to the demonstrated effects on cell lines, cervico-vaginal cells, and cervical explant tissue, lemon and lime juices also exerted inhibitory effects on viability of *Lactobacillus *species associated with normal vaginal flora consistent with earlier reports for the effect of lemon juice on probiotic bacteria [17]. Thus, in combination with direct cytotoxic effects on tissues, indirect effects on microbial flora could lead to vaginitis, a potential cofactor for transmission [23].

There is currently no approved topical microbicide to prevent sexual transmission of HIV, although several products are now in various phases of clinical trials. It is generally agreed that a potential microbicide must be highly efficacious against HIV-1 and demonstrate a lack of toxicity to vaginal flora and cervical-vaginal tissues before being considered as a candidate for evaluation in clinical trials. It must not cause inflammation or sloughing of the vaginal epithelium, and even minor, subclinical toxicity, such as the increased production of pro-inflammatory cytokines, is unacceptable. For microbicide safety evaluations, exposure time, mode of application, and microbicide formulation are key determinants. In response to these safety concerns, several government-and private sector-derived recommendations have been issued for consideration in the development of topical drugs intended to prevent the transmission of sexually transmitted diseases (STD) [24-26].

In the absence of an approved topical microbicide, little is known regarding the clinical relevance (or predictive value) of *in vitro *pre-clinical assays for efficacy or cytotoxicity for candidate products. N-9, cellulose sulfate, Carraguard, and PRO2000 are the only products, for which preclinical data and clinical outcomes can be correlated. A retrospective analysis of pre-clinical N-9 data obtained from several different laboratories showed that *in vitro *cytotoxicity assays were predictive of the clinical results [20]. Correlating preclinical and clinical data from N-9 and other clinically tested products could serve as a basis for early identification of potentially harmful or irritating products using *in vitro *assay systems [20,22,27,28]. The ability to identify which preclinical assays are the best predictors of clinical outcomes could help streamline the preclinical evaluation process and shorten the critical path to development of a safe effective topical microbicide.

Because it is conceivable that vaginally applied juices could be buffered in the vaginal environment via innate factors or the presence of ejaculate, the effects of neutralized juices and vinegar were of interest. Although neutralization of the juices resulted in decreased cytotoxicity in some assays, our data demonstrate that even short pre-exposure of the cells to the cytotoxic effects of naturally acidic juice outweighs any potential antiviral benefits. This suggests that following vaginal application of lemon or lime juice, such short-term damage to the epithelium would likely increase the risk of HIV-1 transmission. In response to reports that women in Asia and Africa were already using lemon or lime juice as microbicidal contraceptives, lime juice was prospectively evaluated in Phase 1 trials [29,30]. It was found that the use of these juices as topically administered preventives is contraindicated based on safety concerns at higher concentrations and predicted low efficacy at lower concentrations. Thus, the results of the presented study demonstrating the *in vitro *cytotoxic effects of lemon and lime juices on the viability of primary lymphocytes, cell lines, explant tissue, and *Lactobacillus sp*. are consistent with the clinical safety results.

## Conclusion

The data from this study and previous reports clearly demonstrate that the use of citrus juices as topical microbicides is potentially more toxic than nonoxynol-9 and thus not recommended for vaginal application.

## Methods

### Test Substances

The test substances lime juice, lemon juice, and household vinegar were purchased and prepared with methods similar to those that women in the field would use for vaginal cleansing. Minor modifications were made to accommodate performance of cell-based assays, as described below. To determine if the acidity of the juices was responsible for the antiviral and cytotoxic effects seen, some evaluations were also performed using the juices and vinegar after neutralization to pH 7.4. Lemons, limes, oranges, and grapefruits were purchased in December of 2005 and May of 2006 at local US chain grocery stores, freshly squeezed, centrifuged at 1,100 × g for 2.5 hours to remove solid particles that could affect assay performance, and stored at 4°C. The stock concentrations of the freshly squeezed juices were defined as 100%. The concentration of juice or vinegar that achieved IC_50_, IC_90_, and TC_50 _values were expressed as percent (%) solution (v/v). The pH of freshly squeezed lemon and lime juice ranged from 2.1-2.4 and 2.2-2.3, respectively. The pH values of orange and grapefruit juices were 3.3 and 3.0, respectively. Apple (pH 3.7) and tomato (pH 3.9) juices were purchased in cans. Weis brand quality distilled white vinegar (5% acidity) was also purchased at a local grocery store. The pH of white vinegar was consistently 2.5, reflecting the standardized nature of the product. For some experiments, lemon juice, lime juice, and white vinegar were adjusted to a neutral pH by adding 10 N sodium hydroxide. The pH range for neutralized lemon juice was pH 7.4-7.6, for neutralized lime juice pH 7.4-7.8, and for neutralized vinegar pH 7.1-7.4. Tomato, grapefruit, orange, and apple juices were centrifuged at 1,100 × g for 2.5 hours to remove solid particles that could affect assay performance and filtered (0.45 μM) prior to use. Working solutions of test substances were prepared and serially diluted by 2-fold, half log_10_, or log_10 _dilution steps starting with a high test of 20-50% juice or vinegar concentration.

The control compounds TAK 779 and AMD 3100 were obtained from the NIH AIDS Research and Reference Reagent Program, Division of AIDS, NIAID and tested at 10 μM and five serial log_10 _dilutions. The following chemicals were purchased commercially: Zidovudine (AZT; Sigma, St. Louis, MO), Penicillin-Streptomycin, liquid (10,000 units penicillin; 10,000 μg streptomycin; Invitrogen, Carlsbad, CA), and Triton-X-100 (Sigma, St. Louis, MO). Nonoxynol-9 was a generous gift from Dr. Gustavo Doncel, CONRAD (Contraceptive Research and Development Program, Norfolk, VA).

### Cells, Bacteria, and Tissues

Cell lines were obtained as previously described [19]. Ectocervical (Ect1/E6E7), endocervical (End1/E6E7), and vaginal (VK2/E6E7) cell lines were a generous gift from Dr. Raina Fichorova of Brigham and Women's Hospital, Boston, MA [31]. *Lactobacillus jensenii *and *L. crispatus *were obtained from the American Type Culture Collection (ATCC 25258 and 33820, respectively, Manassas, VA) and grown in Difco™ Lactobacilli MRS Broth (Difco/Fisher Scientific, Pittsburgh, PA). Human peripheral blood mononuclear cells (PBMCs) were isolated from hepatitis and HIV-seronegative donors by standard ficoll hypaque gradient centrifugation. Transformed cells and PBMC were cultured in complete RPMI (suspension cells) or complete DMEM (adherent cells) containing 10% fetal bovine serum, 2 mM glutamine, 100 U/mL penicillin, and 100 μg/mL streptomycin. Human cervical explant tissues were obtained as previously described [19] without any patient identifiers from normal ectocervix from premenopausal women undergoing routine hysterectomy through the National Disease Research Interchange (NDRI, Philadelphia, PA). All donors were tested for HIV seropositivity, and a pathology report was provided with every shipment, allowing exclusion of tissue with abnormal pathological findings. Experimental protocols had full Institutional Review Board approval and individual patient consent for the use of tissue in research applications.

### Viruses

The following viruses (obtained through the NIH AIDS Research and Reference Reagent Program, Division of AIDS, NIAID) were used: HIV-1_BaL _(CCR5-tropic), [32], HIV-1_IIIB _(CXCR4-tropic) [33,34], HIV-1_JR-CSF _(CCR5-tropic molecular clone) [35], and the primary HIV-1 isolates 92BR020 (Catalog# 1780), and 92UG029 (Catalog # 1650). The origin of the HIV-1 SK-1 strain has been described [36,37]. Chronically infected H9-SK1 and MOLT4/R5/JRCSF were produced in-house.

### Cell-free and Cell-associated Efficacy and Cytotoxicity Assays

HIV-1 Attachment, HIV-1 fusion, and HIV-1 cell-associated transmission inhibition assays were performed as previously described [19]. For cell-based assays, efficacy and cytotoxicity plates were set up in parallel as described elsewhere [19]. Each determination was performed in triplicate and at least three independent experiments were performed, except for neutralized vinegar in the CCR5 cell-associated assay, where only two independent experiments were performed.

### PBMC assays

For the PBMC-based assay, phytohemagglutinin (PHA; Sigma-Aldrich)-stimulated cells from at least two normal donors were pooled and plated in 50 μl at 5 × 10^4 ^cells/well. Cells were exposed to test compounds for 15 to 30 minutes prior to addition of 50 μl of diluted virus stock (HIV-1 92BR020 and 92UG029) at a predetermined titer. Each plate contained no-compound control wells (cells plus virus) and experimental wells (compound, cells, and virus) for two test articles (juices, vinegar, or AZT), evaluated in triplicate wells at nine different concentrations. Cultures were incubated for 7 days and HIV-1 replication in PBMC cultures was determined by measurement of extracellular reverse transcriptase activity as described previously [38]. The HIV reverse transcriptase inhibitor 3'-azido-3'-deoxythymidine (AZT) was used as a positive control for all PBMC assays.

### Cytotoxicity Assays

Cell viability was determined using CellTiter 96^® ^AQueous One Solution Cell Proliferation Assay (Promega, Madison, WI), LIVE/DEAD^®^, or VYBRANT™ kits (Molecular Probes, Invitrogen). The CellTiter 96^® ^AQueous One Solution Cell Proliferation Assay contains a tetrazolium compound [3-(4,5-dimethylthiazol-2-yl)-5-(3-carboxymethoxyphenyl)-2-(4-sulfophenyl)-2H-tetrazolium, inner salt; MTS] and an electron coupling reagent (phenazine ethosulfate; PES). PES has enhanced chemical stability, which allows it to be combined with MTS to form a stable solution. MTS is bioreduced by cells into a colored formazan product that is soluble in tissue culture medium [39]. This conversion is presumably accomplished by NADPH or NADH produced by dehydrogenase enzymes in metabolically active cells [40]. The LIVE/DEAD^® ^Viability/Cytotoxicity Kit for mammalian cells is based on the simultaneous determination of live and dead cells with two probes that measure recognized parameters of cell viability-intracellular esterase activity and plasma membrane integrity, using the dyes calcein AM and ethidium homodimer (EthD-1). The polyanionic dye calcein is well retained within live cells, producing an intense uniform green fluorescence in live cells. EthD-1 enters cells with damaged membranes and undergoes a 40-fold enhancement of fluorescence upon binding to nucleic acids, thereby producing a bright red fluorescence in dead cells. EthD-1 is excluded by the intact plasma membrane of live cells. In the Vybrant^® ^Cytotoxicity Assay Kit, damaged and dying cells release glucose 6-phosphate into surrounding medium. The glucose 6-phosphate is detected by an enzymatic process that leads to the reduction of resazurin into red-fluorescent resorufin. Each determination was performed in triplicate. Three separate experiments were performed for natural lemon juice. For neutralized lemon juice, natural and neutralized lime juices, and vinegar, one experiment was performed in triplicate.

### *Lactobacillus *Toxicity Assay

In order to assess the potential effect of vaginal application of the juices or vinegar on the viability of H_2_O_2_-producing bacteria (*Lactobacillus jensenii *and *L. crispatus*, ATCC 25258 and 33820, respectively) associated with vaginal flora, a standard antimicrobial broth dilution assay was used as previously described [19].

### HIV-1 Infection of Cervical Explants

Explants were activated for 2 days in complete DMEM containing 5 μg/mL phytohemagglutinin-P (PHA; Sigma, St. Louis, MO) and 100 U/mL human interleukin-2 (IL-2) (Roche, Indianapolis, IN) and infected as previously described [19,41]. Briefly, on day 3 after stimulation, tissues were pretreated with test substances for 1 hr, and explants were infected overnight, followed by 5 washes with PBS. Culture medium was added back, and supernatants were harvested every 3-4 days over a 14 day period and stored at-70°C. Viral replication was determined by HIV-1 p24 ELISA (Beckman Coulter, Miami, FL).

Viability of the explants and assessment of microbicide toxicity were quantified as previously described [19,41]. Briefly, cervical explants (3-mm diameter) were incubated with or without test article in complete DMEM. For comparison, a tissue control was used that was incubated in medium alone. For toxicity determinations, after exposure to products for 2-24 h, explants were washed 5 times in PBS, and then immediately cultured in complete DMEM containing MTT (250 μg/mL) for an additional 2 h at 37°C. Tissues were then placed in 200 μL absolute methanol for a minimum of 24 h (protected from light), and after removal from the methanol, allowed to air dry for a minimum of 48 hours. Tissue viability was determined by dividing the optical density of the formazan product (570 nm) by the dry weight of the explant. The effect of each product on tissue viability was determined by comparing the viability of the treated explants to the untreated tissue control.

### Data Analysis

The IC_50 _(concentration of test compound resulting in a 50% decrease in virus growth compared to a virus control that included only cells, virus, and culture media), TC_50 _(concentration of test compound resulting in 50% of cell viability as compared to a control that included only cells and culture media), and MIC_50 _(concentration of test compound resulting in 50% bacterial growth compared to a control that received only bacteria and culture media) were calculated as previously described [19]. The therapeutic index (TI) was calculated by dividing the TC_50 _by the IC_50_. Individual assays used triplicate measurements that were averaged, and between 2-17 assays were run using each method and product, unless otherwise specified. Virus growth (p24 pg/mL) was calculated for cervical explant assays testing HIV-1 inhibition of lemon juice (5 & 20%) and lime juice (5%) compared to the no treatment condition (i.e., virus control). Proportional measurements (% virus growth and % cell, bacterial, and explant viability) were reported using mean and standard deviation, whereas IC_50_, TC_50 _and p24 pg/mL concentrations were reported using median and inter-quartile range. The % bacterial viability following exposure to neutralized and natural products were compared by one-way Analysis of Variance (alpha = 0.05).

## Competing interests

The authors declare that they have no competing interests.

## Authors' contributions

CLS initiated the study, coordinated the technical team, wrote the first manuscript draft, and presented the data at meetings. BS and KM prepared the test articles and performed the attachment, fusion, cervico-vaginal cell line, and *Lactobacillus *assays. MCO and MM performed the cell-associated and PBMC assays. MJ performed the cervical explant assays, and LND carried out some of the *Lactobacillus *assays. NRH analyzed the data and generated publication-quality figures. JC and BES-B as Principal and Co-Principal Investigator of the contract provided overall leadership on the design of the experiments and study. BES-B also guided writing of the manuscript. All authors read and approved the final manuscript.
